# The Scandal of "Hospital Scandals"

**Published:** 1900-11-24

**Authors:** 


### The Institutional Workshop.

### THE SCANDAL OF "HOSPITAL SCANDALS."

On more than one occasion recently we have entered our protest against the habit which certain newspapers have of heading every complaint against any of the hospitals as "Another Hospital Scandal." The two chief offenders in the metropolitan press are the *Star* and the *Echo*, and the injustice and evil arising from the practice in their case are increased by the want of knowledge exhibited and the comments made. Thus severe attacks have been made on St. Bartholomew's Hospital in the *Star* during the last month, and the cases of complaint in regard to this hospital have been made a peg on which to hang the assertion that it is not right to ask the public to give more money to the London hospitals when the abuses complained of prevail at St. Bartholomew's. As a matter of fact, of course, the Treasurer of St. Bartholomew's Hospital has never asked for or received any money from the public towards the support of that institution, which is an endowed hospital with ample funds to defray the cost of all the work done. Generally, we may repeat what we have before stated, that if all the cases of mistake or complaint which arise in a given year are totalled up, they do not amount to a thousandth part of one per cent. of the whole number of cases handled by the metropolitan hospitals collectively in any period of twelve months. To state this fact is to reveal the injustice and unfairness of the comments and head lines which too frequently appear in the newspapers. If the editors would consider for a moment, they would be forced to admit that they might well regard themselves as fortunate were it possible for them to claim that of all the matters and papers which pass through their hands in a given year not one thousandth of one per cent. of the whole go wrong. We are confident that the chief editors of the papers in question do not realise the evil which is wrought, or the injustice of the practice of which we complain, or they would give instructions which would terminate this grave abuse of the functions of a reputable organ of public opinion.

### The True Picture of a Hospital.

It has been our good fortune in the last few months to make a thorough inspection of several of the metropolitan hospitals, general and special. Our visits were paid without any previous notice to the authorities, and were made at all sorts of hours, so that we were able to see the work proceeding and to ascertain the condition of administration of the whole institution. A fortnight ago, on a miserably wet, cold day, when the streets of the East End of London looked at their worst, and filth was everywhere the most prominent feature, we made our way to two hospitals in the East End. At the first, a relatively small institution, which looks after maternity cases in poor homes, and admits a certain number of mothers for treatment, we found everyone cheerful and alert. The work was being carried on in the most excellent way, and an inspection of the institution tended to cheer the visitor and to make him grateful for the self-denying, conscientious service which was being rendered by the staff and the nurses to the very poorest of the poor in their hour of direst necessity. Passing on to a large hospital devoted to children we found the same general state of efficiency throughout the buildings. In the wards, where the older children were under treatment, we found the occupants bright and cheerful and happy, although not a single inmate was free from suffering, and all were undergoing treatment for very serious maladies. In one large ward, where the younger children were placed, no child being probably more than five years of age, there were some thirty little mites, every one of whom presented the appearance of retarded development, pale and emaciated features, and all the signs of neglect and semi-starvation. Yet there was not a child which was not perfectly contented with its surroundings, and, childlike, each inmate seemed to forget the miseries of the past in its present atmosphere of kindness and care, combined with perfect order, cleanliness, and the closest attention to everything calculated to restore the child to a normal condition of health. Visits like these impress us with the feeling that if it were possible to get those members of the community who have little or nothing to do, who suffer from ennui, who regard life as a burden and a misery, to visit an institution of this kind, and to strive to become familiar with the causes which underlie the suffering represented by the patients in the beds, and the means which are brought to bear with so much success to alleviate that suffering under the most trying circumstances, the benefits, both moral and physical, to the visitor would be immeasurable.

In view of an experience like that we have just recorded, it should be manifest to every editor that the scandal of "hospital scandals" lies in the fact that there should be one responsible conductor of a metropolitan journal, who, instead of sending his representative into our hospitals to see and to record what is actually being done there on behalf of the poor and suffering, contents himself with permitting any reporter to fasten on to some apparent mistake in treatment, or some accidental omission to correctly diagnose a disease in difficult circumstances, and to use it as a handle to make the work of those responsible for our hospitals more and more difficult to accomplish successfully.

### A Word for the Workers.

It is a surprising fact, but it is a fact, that there are very many people who apparently fail to realise the enormous difficulties under which the work of the hospitals is so successfully done in a vast community like London. It should not be forgotten that during every minute of every hour of every day in every week throughout the year hospital doors are always open, and any sufferer, however humble or however high, may be taken to any one of these institutions with the assured certainty that the sufferer will there receive much more rapid and much more effectual aid and treatment than can be secured anywhere else. It is matter for great thankfulness that the army of good men and women who devote their lives to the alleviation of suffering never decreases, but year by year the numbers who are attracted to the work, and who give wholly of themselves for its efficient performance, continues steadily to increase. When everything has been said that can properly be said by way of criticism, there remains an aggregate of solid, successful, Christlike work to the credit of our hospitals which should silence the carpers; for its volume is so overwhelming as to make it matter for thankfulness and surprise that the mistakes which occur in the course of a year's work at these grand institutions are so relatively unimportant and so marvellously few in number.

